# The Case of Miss Buchanan

**Published:** 1887-12-03

**Authors:** 


### THE CASE OF MISS BUCHANAN.

We regret that we do not think the further correspondence the mother sends on this subject suitable for insertion in The Hospital, and in answer we can only repeat our previous statement, that the whole case has a legal and not a medical bearing, and consequently should be placed in the hands of a solicitor. The question is not now one of good or bad nursing at home or elsewhere; but whether the patient is or is not rightfully detained.

